# Evaluating step-down, intermediate care programme in Buckinghamshire, UK: a mixed methods study

**DOI:** 10.1186/s12889-023-15868-5

**Published:** 2023-06-06

**Authors:** Fani Liapi, Angel Marie Chater, Tina Kenny, Juliet Anderson, Gurch Randhawa, Yannis Pappas

**Affiliations:** 1 Institute for Health Research, University of Bedfordshire, Luton, LU2 8LE UK; 2 Institute for Sport and Physical Activity Research, University of Bedfordshire, MK41 9EA Bedford, UK; 3 University College London, Centre for Behaviour Change, WC1E 7HB London, UK; 4 Buckinghamshire Healthcare NHS Trust, Aylesbury, HP21 8AL UK; 5 Buckinghamshire Health and Social Care Academy, Aylesbury, HP21 7Q UK

**Keywords:** Intermediate care, Step-down care, Person-centred care, Rehabilitation, Patient experience

## Abstract

**Background:**

Intermediate care (IC) services are models of care that aim to bridge the gap between hospital and home, enabling continuity of care and the transition to the community. The purpose of this study was to explore patient experience with a step-down, intermediate care unit in Buckinghamshire, UK.

**Methods:**

A mixed-methods study design was used. Twenty-eight responses to a patient feedback questionnaire were analysed and seven qualitative semi-structured interviews were conducted. The eligible participants were patients who had been admitted to the step-down IC unit. Interview transcripts were analysed using thematic analysis.

**Findings:**

Our interview data generated five core themes: (1) “Being uninformed”, (2) “Caring relationships with health practitioners”, (3) “Experiencing good intermediate care”, (4) “Rehabilitation” and (5) “Discussing the care plan”. When comparing the quantitative to the qualitative data, these themes are consistent.

**Conclusions:**

Overall, the patients reported that the admission to the step-down care facility was positive. Patients highlighted the supportive relationship they formed with healthcare professionals in the IC and that the rehabilitation that was offered in the IC service was important in increasing mobility and regaining their independence. In addition, patients reported that they were largely unaware about their transfer to the IC unit before this occurred and they were also unaware of their discharge package of care. These findings will inform the evolving patient-centred journey for service development within intermediate care.

**Supplementary Information:**

The online version contains supplementary material available at 10.1186/s12889-023-15868-5.

## Background

Healthcare systems worldwide are under pressure as a result of population growth, aging, patients’ complex needs, and the emergence of patient-centred care [1, 2]. COVID-19 added more pressure to healthcare systems. In the UK, the COVID-19 pandemic presented the National Health Service (NHS) probably with the most significant challenge in its 70-year history [3]. To relieve pressure on inpatient services, NHS England has developed the national Patient Discharge Services Framework, to support hospital discharges and streamline the transition from the hospital to the community [4].

Discharging people from hospital to community has become increasingly complex and the number of patients requiring additional support is increasing [5]. Among hospitalised people, some cannot be discharged to their homes, as the arrangements for their health and social support (e.g. carers, nursing home beds) have not been completed. Delayed discharges for those who are medically fit to be discharged, increase the pressure in acute care and the risk for healthcare-associated infection (HAI) [6, 7]. To address these issues, the NHS focuses on boosting ‘out-of-hospital’ care [8]. The National Institute for Health and Care Excellence (NICE) recommended the commissioning of integrated services that help people to recover, regain independence, and return home safely [9].

Intermediate care (IC) aims to bridge the gap between hospital and home, enabling continuity of care and the transition of patient care to the community [10, 11]. They are broadly defined as ‘*…that range of services designed to facilitate the transition from hospital to home, and from medical dependence to functional independence, where the objective of care is not primarily medical, the patient’s discharge destination is anticipated and a clinical outcome of recovery or restoration of health is desired’* [12]. There are four broad service models of intermediate care; bed-based services, community-based services, crisis response services, and reablement services [13, 14]. In the UK, the bed-based intermediate care services are provided in an acute hospital, community hospital, residential care home, nursing home, standalone intermediate care facility, independent sector facility, local authority facility, or other bed-based settings are time-limited to stays that are no longer than six weeks [14, 15].

Intermediate care is a healthcare pathway that is integrated across health and social care. Integrated working has been promoted in national policy [8, 15, 16]. It promotes person-centred care; a type of care that focuses on the needs of individuals, ensures that people’s preferences and needs guide clinical decisions, and provides respectful and responsive care [8, 17, 18]. It is expected that integrated person-centred care will provide improved healthcare to patients with complex health needs, which in line leads to patients’ positive experience with the healthcare system [19].

Responding to the above priorities, Buckinghamshire Healthcare NHS Trust, an organisational unit within the NHS in England, developed a step-down IC service with the aim to provide bed-based care for Buckinghamshire patients at the end of their episode of acute care, providing support and the encouragement of independence, whilst patients are waiting for their home package of care or onward placement. The service opened as a bedded care facility on the 31st of January 2022 and closed on the 27th of May 2022. During this time, it provided accommodation and personal care for up to 22 patients at one time. The bedded area was situated in a nearby hotel that was equipped to accept patients.

A recent review of global published evidence, examining the effectiveness of intermediate care, reported positive outcomes on patients’ functional ability and resulted in reductions in hospital utilisation [10]. Other outcomes, including improvements in patient satisfaction and quality of life, were also reported but the evidence was limited. Furthermore, the review was focused on intermediate care interventions, including transitional care delivered in hospital and community settings, intermediate care delivered at home, and bed-based intermediate care interventions. Fourteen studies, out of 133 included in the review, reported the effectiveness of bed-based intermediate care. However, none of these studies explored the patients’ lived experience and satisfaction with the care.

The present study is part of an audit of a step-down IC service in Buckinghamshire, UK. Overall, the evaluation was aimed at exploring the implementation and impact of the step-down IC service, and to inform other intermediate care initiatives in the NHS. The evaluation used a mixed methods approach consisting of qualitative and quantitative methods. More specifically, the current study aims to explore the lived experience and satisfaction of people receiving care in the step-down IC service. The findings of this study would provide a better understanding of the needs and preferences of those receiving care in step-down IC services, as well as the satisfaction levels they experience. This information can be used to inform policy decisions and the design of more effective care services, both in the UK and abroad.

## Methods

### Setting

The unit was a 22 bedded step-down IC service in Buckinghamshire, UK. The unit opened as a bedded care facility on the 31st of January 2022 and closed on the 27th of May 2022 to support winter pressures and provide accommodation and personal care for up to 22 patients at one time. The service was set up to support hospital discharges.

### Design

A mixed-methods study design was employed to explore the experiences of the patients. Responses to a patient feedback questionnaire were analysed and qualitative semi-structured interviews were conducted. Hence, this mixed methods study evaluates [1] the patient experience and satisfaction using a feedback questionnaire and [2] the patient experience and satisfaction using qualitative methods. The results from the questionnaire were analysed and used as input for an in-depth analysis of the patient experience and satisfaction with care at the service. The consolidated criteria for reporting qualitative research (COREQ) checklist [20] were completed to ensure all relevant items for reporting qualitative research were included (Supplementary file 1).

### Participants

A purposeful sampling method was used to obtain variation in the age and gender of the patients. The service matron identified the potential participants, who were able to communicate and provide consent. In addition, the matron facilitated the recruitment, as she introduced the study to the participants and she requested their verbal consent to be approached by the researcher. A total of 7 participants (5 females; 2 males) participated in the interviews, which took place in May 2022. Participants at the time of the interview, were between the ages of 54–83 years old (*M* = 71.14 years; *SD* = 11.48). Participants’ characteristics cannot be included as they can lead to the identification of the participants. Recruitment ceased when data saturation was reached.

### Data collection

The patient feedback questionnaire was provided to the patients by the nursing staff before their discharge from the IC setting. When the evaluation of the service started, the completed questionnaires have been made available to the researchers for analysis (Supplementary file 3).

Individual face-to-face, semi-structured interviews were conducted with patients. Interviews took place at the intermediate care site by FL. The interviews lasted between 15 and 30 min. Before the interviews, the participants were fully informed about the interview process. The matron provided a brief description of the study to the participants and gained their verbal consent to be approached by the researcher. The researcher provided a participant information sheet to the participants, explained the process, and answer any questions that were raised by the participants. The participants were informed that the interview will be audio-recorded and they were reassured that any information they share would be kept anonymous. Written, informed consent for participation in the study was signed by the participants prior to the interviews. The study was aimed to understand patient experience from admission to discharge. Therefore, the interview guide was developed in a way that aims to follow the patients’ journey throughout the intermediate care pathway. The interview guide was based on the relevant literature and our clinical and administrative experience in the NHS (Supplementary file 2). The results from the questionnaires were also informed the development of the interview guide.

The study was approved by the Institute for Health Research Ethics Committee (IHREC) of the University of Bedfordshire, UK (Application No: IHREC940; date of approval: 05 May 2022). Informed consent was obtained from all participants involved in the study.

### Data analysis

**Fig. 1:**
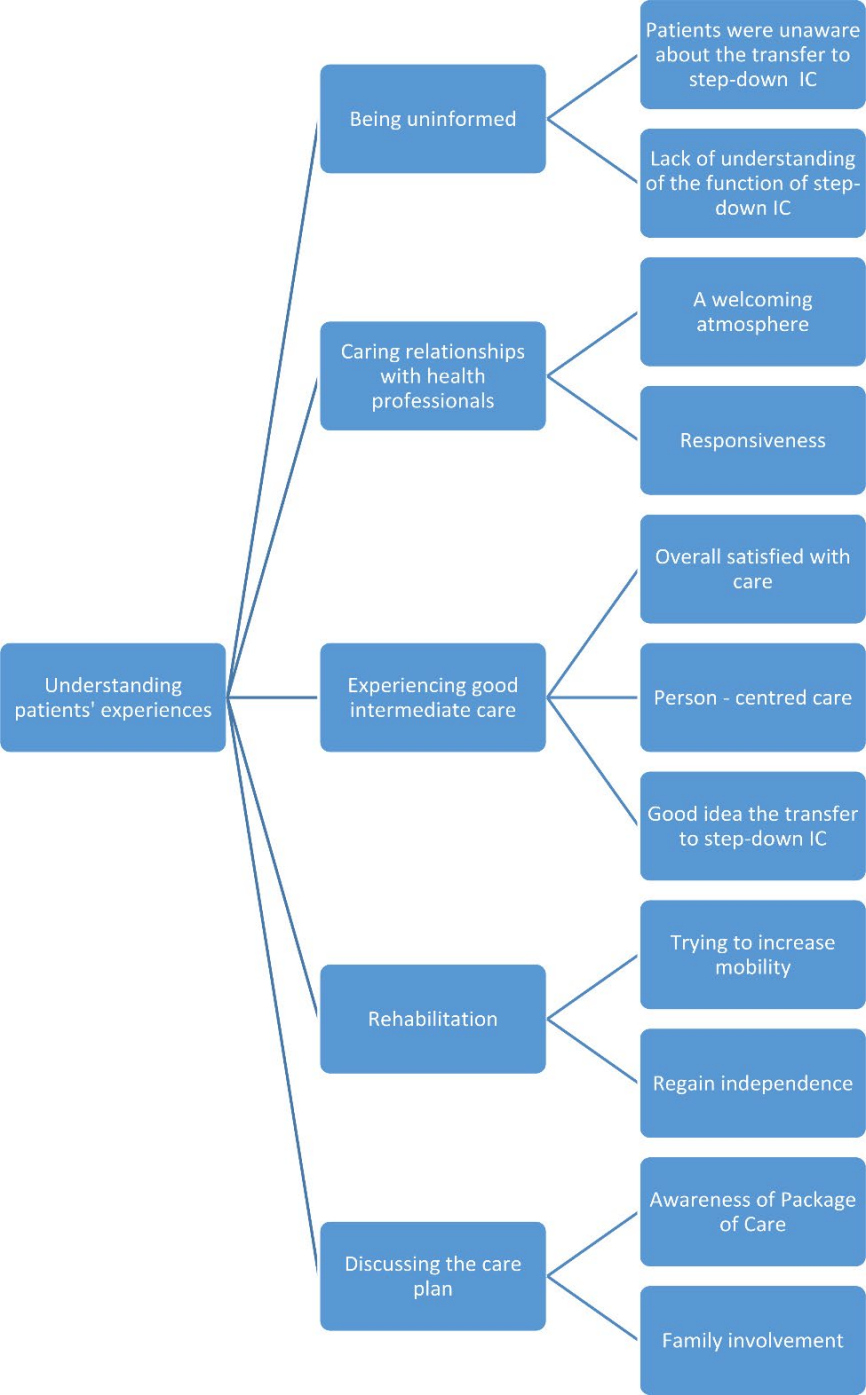
The coding tree of the key themes identified from the data investigating patients’ experiences and satisfaction with admission to step-down IC

The interviews were audio-recorded and transcribed verbatim by FL. The NVivo software [21] was used to analyse the interviews. After familiarisation with the data, all transcripts were coded line by line by a single researcher (FL) following an inductive approach. Reflexive thematic analysis was used to analyse the transcripts, identify sub-themes and group them together under sub-themes, which were discussed with the wider team [22]. Data saturation was reached by the 5th interview. Braun and Clarke [23] stress that data saturation cannot be tied to the number of interviews. Also, they argue that coding quality in reflexive thematic analysis stems not from consensus between coders, but from depth of engagement with the data and situated, reflexive interpretation [23]. Reflexivity is a fundamental part of ensuring the transparency and quality of qualitative research [24]. Interviewer’s reflexive notes which had been made throughout the interview process were taken into account on data analysis process. The themes were derived from the dataset and the interpretive process of the researcher. The process of coding and developing themes was descriptive (representation of what the participants said) and interpretive (consideration of less direct evident patterns based on researcher’s experience and interaction with the participants). A coding tree was produced to assist with visualisation of the findings (see Fig. 1). We also provide a descriptive statistical analysis of the feedback questionnaire. The questionnaires were coded and analysed using SPSS software. [25].

## Findings

A structured questionnaire was developed by the providers to assess patients’ experiences of the IC. The patient experience questionnaire explored issues such as providing information about the service, family involvement, and satisfaction with the provided care and staff. 28 questionnaires were returned to providers and included in the analysis. There are no available data that inform the total number of questionnaires distributed to the patients.

The findings from the patient questionnaire show that patients were happy with most aspects of the step-down IC. Twenty-seven patients (96.4%) responded that they felt safe and cared for in the step-down IC, and one patient responded that they would have liked more help. 75% of those who returned the questionnaire indicated that all of the staff had gone above and beyond, while 17.9% of the respondents indicated a particular person who exceeded their expectations. Just over half of the respondents (57.1%) reported that they knew what to expect in terms of their ongoing discharge, while 28.6% of the respondents were unsure about their discharge plan. In response to the question: “Have your family felt they received enough information about IC and your discharge?”, 46.4% of the respondents reported that their family felt included, 32.1% stated that their family would like more information, and 10.7% expressed that their family was unsure about what was happening. However, the data reveals that just over half of the patients (57.1%) have not been given any information about step-down IC before their arrival.

The thematic analysis led to the identification of five main themes, which describe the experiences of patients regarding the service they received from the step-down IC. The main themes are: *(1) Being uninformed, (2) Caring relationships with health practitioners, (3) Experiencing good intermediate care, (4) Rehabilitation, and (5) Discussing the care plan.* These core themes were structured from subthemes that further describe the patients’ experiences. A visual representation of the themes can be seen in Fig. 1.

### Theme 1: being uninformed

#### Patients were unaware about the transfer to the step-down IC

In their accounts, patients expressed that they were unaware about their transfer from the hospital to step-down IC. They mentioned that they have not been told why they are transferred to step-down IC.

*“All I know it is that they just brought me here. They just told me that they will transfer me, they put me on a wheelchair, and they pulled me over. All of the sudden. […] I wasn’t pre-warned or anything like that.” (Participant 2)*

*“I didn’t know I was coming here. It was all very strange. […] I was a bit confused by it all, to be honest.” (Participant 6)*

Although, one interviewee, commented that they were given some information, but not until the day of the actual transfer.

*“I was given a sheet of paper and I wasn’t told too much about it… was overflow COVID. […] Yes, I was given some information. The hospital didn’t really tell me until it was almost that day. So I wasn’t warned a week beforehand.” (Participant 4)*

#### Lack of understanding of the function of the step-down IC

Participants stated that they were not given information about the function of the step-down IC. Some participants were provided with some information about the facilities. Therefore, participants were unaware of why they have been transferred to step-down IC.

*“They told me, I’d have the room on my own and my own bathroom. And they told me that it was a nice place to go into which it is.” (Participant 1)*

*“I still don’t know. I don’t know why I had to come here. All I was told was for an extra bit of rest.” (Participant 3)*

### Theme 2: caring relationships with health practitioners

#### A welcoming atmosphere

The majority of the participants expressed their satisfaction with the nursing team. They highlighted that the nursing team offers them a friendly, warm, and caring environment.

*“They’re just so friendly, warm, caring. I can’t say enough about them.” (Participant 3)*

*“Lovely. Everybody’s been great. We’ve had a good laugh time at night-time with the nurses. They been absolutely fantastic. They’ve all been great.” (Participant 7)*

#### Responsiveness

Participants stated that the members of staff were available and responsive to meet their needs and answer any queries that they may have.

*“I couldn’t wish for anybody better. So, you know if you need anything, which I try not to…. But they are so happy and if I want to know something they would tell me.” (Participant 3)*

*“They are on your doorstep, you know… I‘ve got this remote and if you want anything, you press that button and they are here quickly as flash.” (Participant 6)*

### Theme 3: experiencing good intermediate care

#### Overall satisfied with care

In their narratives, participants expressed their satisfaction with their stay at step-down IC. They described positively their experience of care in IC and they highlighted that they had no complaints.

*“Since I’ve been here, it’s been really well for me. […] It is really fine. You wouldn’t wish for anything better.” (Participant 3)*

*“They’ve been treated me very well. So I’ve got no complaints. […] They are very nice. Excellent. So I can’t complain about anything. I’ve been looked after.” (Participant 6)*

One participant stated that he had two negative experiences during their stay at step-down IC. The first one was the lack of supplies, specifically clean sheets, and the second was that they had to repeatedly ask to be seen by the pain team, because of his pain issues.

*“We ran out of sheets one day, a couple of weeks ago. You think no hospital run out of sheets, but this isn’t the hospital, they run out of things here. And then any sudden panic try and get them so I’m not sure whether that’s an ordering issue, whereas somebody here should realize the sheets are running out and should have ordered them in time. Also, because I was having problems with my pain, I’ve asked for the pain team three times.” (Participant 4)*

#### Person-centred care

Participants stated that they received care based on their individual needs. From the following quote, it seems that step-down IC made the appropriate arrangements to meet patients’ individual needs and speed recovery.

*“I came in here malnourished. They’ve given me exactly what I’ve asked. Normally you have a hot meal for lunch and then a sandwich or something for dinner or supper. But after I spoke to my dietician, I spoke to them and now they know me. They give me a hot dinner for lunch and supper. And when I woke up at the night, my dietician told me: “If you wake up and you feel hungry, ask the nurses to get something to eat, like a sandwich, because they’re 24 hours a day. They’ve got to provide it and they’ve done it.” (Participant 4)*

Participants compared the communication between the different departments in the hospital and between the different teams in the step-down IC. A participant commented that they received more coordinated care in step-down IC, compared with the care they had received in the hospital.

*“My only downside, is not about IC, is about the hospital, is that one department doesn’t kind of talk to the other. […] I mean, different departments don’t speak to each other. […] I think it’s better here, a lot better here. Because this is like a small hospital. This is a bit of a smaller community. It’s a lot easier. I feel the care is more co-ordinated in IC.” (Participant 1)*

#### Good idea the transfer to step-down IC

Participants stated that their transfer from the hospital to step-down IC was a good idea. They appreciated that they were fit enough to leave the hospital and free up beds for more critical patients. Some participants expressed their desire to return to their homes, but they were happy with their stay in step-down IC until their package of care would be arranged.

*“I was good to be moved from the hospital to here because I wasn’t dying. I was just recuperating. So doesn’t matter where I am, I can receive some care here, so they put me here to free up the bed, which makes sense because there are more critical patients. […] It’s a good idea to put me here because as I said, it frees up a bed for more critical patients who are coming in. So I thought yeah, good chance as they’re shutting this down at the end of the month.” (Participant 4)*

*“Obviously this must be the transition. This was been a move here because I’m well enough to go home now. One of the reasons why I am here I think is to look after myself. And obviously this is a transition, just waiting now.” (Participant 2)*

### Theme 4: rehabilitation

#### Trying to increase mobility

Participants stated that they are working towards increasing their mobility during their stay in step-down IC. Also, they expressed that they were safe while they were trying to increase their mobility because they knew that there was help available.

*“I don’t need assistance to go to the toilet or walk. I am ok with the stairs, as long I take it easy now. One thing that I can’t do at the moment, because I tried it here, I can’t go up the steps carrying something. I have to remember that. No messing about that one.” (Participant 2)*

*“They gave me that blue thing (a stretch band), so I can stretch out my triceps, only got that three days ago. So I can stretch my triceps a bit sore now because that’s the first time I’ve used them. That’s useful. […] That’s why the doors are open all the time. I always ask the door to be kept open. So if I fall on the floor… I can’t actually… my body is very weak. I am in bed, my muscles have atrophied.” (Participant 4)*

#### Regain independence

Participants expressed that they were more independent after their stay at step-down IC. Although, they appreciated that they needed to be careful when they would return to their homes.

*“I am more independent. I mean, I use this (walking assistant). I use it all the time. Because one slip and I could be back here. And if I’m asked to do something, I would do it.” (Participant 3)*

*“I think I am a little bit more independent, I’m a bit worried at the night time when you will be on your own at home” (Participant 7)*

### Theme 5: discussing the care plan

#### Awareness of package of care

The majority of the participants expressed that they have not been given enough information about their package of care. Also, they commented that they were worried about being told about their package of care suddenly, as it happened with their transfer to step-down IC.

*“I don’t know when I’m going out of here apart from the fact I know this place is shutting down on the 31st. […] I’m aware of the plan broadly, but I haven’t been involved. They haven’t told me specifically. They’ve said: “These are the options.” They gave me a few options that they’re looking at. I’m waiting to hear back from them. It worries me the fact that they could just come in here and say: “Right, we are moving you now” like exactly what happened before. I’d rather be said: “Look, this is going on.” And I haven’t had much of that. (Participant 4)*

*“I don’t know. It’s supposed to be carers, coming in, three times a day” (Participant 7)*

One participant stressed that they were informed about their package of care and they knew what to expect after their discharge from the step-down IC.

*“I know what to expect. And my equipment that I need is already in the house. They put equipment in for me.” (Participant 1)*

#### Family involvement

Participants stated that their families were informed about their care and their package of care from them. Participants were not sure if their families had been informed about their care by the step-down IC’s staff.

*“I keep them involved. I’ve got a group set up on WhatsApp, and I informed my family. […] So my family is aware. […] I need to ask them. They may have got some information from the staff. I don’t know. I’ve told staff, and the nurses, that if anyone asks, give them more information. And so I’ve done that. (Participant 4)*

*“Yes. I told everybody on my phone.” (Participant 7)*

Although, one participant stated that her husband had concerns about her package of care, but all of his questions had been answered by the discharge nurse.

*“My husband has concerns, they’ve answered all his questions.” (Participant 1)*

## Discussion

This mixed-methods study showed that the patients’ overall experience with the admission to the step-down IC care facility was positive. The analysis of the interviews generated five core themes: (1) “Being uninformed”, (2) “Caring relationships with health practitioners”, (3) “Experiencing good intermediate care”, (4) “Rehabilitation” and (5) “Discussing the care plan”. When comparing the quantitative to the qualitative data, these are consistent. Patients expressed their experiences about being uninformed, their transfer to the service, and not having enough information about their discharge package of care. Patients also reported some positive aspects of their care. Various factors influence the patients’ experience such as; being unaware about their transfer to the service, staff’s performance, rehabilitation support, and being unsure of their care package.

Patients reported that they were largely unaware about their transfer to the step-down IC unit before this occurred. The continuous transfer of patient information between healthcare settings is crucial for the effective continuity of care [26]. Therefore, hospital discharge should include the involvement of hospital staff in providing useful and timely information about any transfer [27, 28]. Participants’ experiences of lack of information are voiced in other studies that found patients being unprepared to be transferred to a step-down IC facility [29–31]. Previous studies comment on two factors that lead to patients experiencing undesirable feelings about their transfer to a step-down IC facility: (a) clinical staff’s lack of understanding of what a step-down IC service involves [32] and (b) patients not having the opportunity to ask questions about their future care [33]. These factors can have a detrimental effect on the patient experience and may lead to a sense of uncertainty and anxiety. Furthermore, the lack of information and understanding can lead to a feeling of being isolated and unheard. Therefore, it is important to ensure that the transition from acute care to a step-down facility is done in an informed and supportive way. Consequently, it is imperative for commissioners and managers of IC step-down programmes to provide not only more information to the patients [30] but to keep the workforce well attuned with the structure and the reasoning of the IC, step-down programmes. By understanding the structure and reasoning of the IC step-down services, healthcare professionals will be able to explain what a step-down IC facility involves to patients in a way that will help them to understand their transfer and future care.

In addition, patients and their families were also unaware of their discharge package of care. Previous research has established that patients and carers generally have an inadequate level of health literacy [34], leading to more attention being paid to continuing to medicalise the patients rather than making them being independent and confident in how to self-care. These issues could be further compounded by the lack of a consistent discharge planning process. The lack of necessary information, guidance, or support to transition home safely and with confidence might lead to increase readmission to hospital rates. Therefore, healthcare providers should ensure that patients have access to the information and assistance they need to successfully manage their health.

Patients highlighted the supportive relationships they formed with healthcare professionals in the step-down IC. Previous studies exploring the effectiveness of intermediate care, have emphasised that quality support from healthcare professionals is a factor that contributes to overall patient satisfaction and reduction in readmission rates [33, 35, 36]. In addition, patients commented positively on the responsiveness of healthcare professionals. Being available to patients increases the chances for the patients to discuss their needs or health concerns. This is in agreement with patient-centered care, where healthcare professionals listen to patients’ concerns, understand their worries, and involve patients in the decision-making about their healthcare plan [37]. Shared decision-making is a collaborative process through which patients and healthcare professionals make healthcare decisions together using the best available evidence and taking into account the patient’s preferences and personal needs [38]. For instance, shared decision-making might involve a healthcare professional discussing the benefits, risks, and side effects of treatments with a patient, helping them to make an informed decision about the best course of action. This approach increases the patient’s sense of control and autonomy, which can lead to better overall health outcomes and a greater sense of wellbeing. Furthermore, it helps to build trust between the patient and the healthcare provider, creating a stronger relationship and a better overall experience. Therefore, shared decision making can be seen as a way of improving the quality of care by ensuring that patients’ needs, values, and preferences are taken into account in healthcare decisions.

Ten years ago, in a Health Foundation funded project to improve patient flow, Sheffield Teaching Hospitals developed the concept of Discharge to Assess (D2A). The D2A care model describes the intermediate care pathways which provide ‘step-down’ care following an acute hospital stay. One of the pathways concerns the provision of short-term rehabilitation in a temporary bedded setting [39]. Against this background, the examined service was implemented with the aim to reduce the length of stay for people in acute care, improve patients’ experience of receiving healthcare, and reduce the overall cost of provision. A recently published scoping review examining the effectiveness of intermediate care reports that the most frequently reported outcome is the reduced length of stay or readmission in acute care [10]. This result suggests that intermediate care has great potential to reduce the burden of acute care on the healthcare system. Furthermore, these findings are consistent with other studies that have found intermediate care to be an effective way to reduce hospitalizations and a cost-effective option for the treatment of patients who are medically fit to be discharged from an acute setting [40]. The service under evaluation was implemented for four months due to limited funded. During the Covid-19 pandemic, extra funding was available to support discharge. The NHS Confederation and NHS Providers [41] stress in their assessment that D2A is a cost-effective policy and it should become the default process for hospital discharge – supported by additional funds to assist with post-discharge care costs. In light of the above evidence, it is important for integrated care systems to develop plans to support, embed, and improve discharge models.

The majority of people interviewed had functional deficits related to mobility, therefore the rehabilitation that was offered in the step-down IC service was important in increasing mobility and regaining their independence. Participants expressed that they were working towards increasing their mobility during their stay in the service and they had increased their independence. The interviews in this study revealed that rehabilitation plans could benefit from clearer goal-setting, about maintaining and improving mobility, and communication. A recent study argues that goal-setting is a key characteristic of modern rehabilitation, as it is considered more person-centred, boosts patients’ motivation and psychological adaptation, and requires strong communication between healthcare professionals, patients, and families [42]. Considering that person-centred care is the first principle that underpins the delivery of intermediate care [43], goal-setting should be considered as a step in tailoring rehabilitation to patients’ needs. However, not all patients may be ready or willing to set goals for themselves. In some cases, patients may be dealing with complex emotions and may need support in other areas, such as increasing their independence levels, before they are able to set goals. Healthcare professionals are required to be aware of these limitations and be prepared to adjust their rehabilitation plans accordingly.

Public and patient involvement (PPI) in healthcare has been shown to increase the quality, relevance, and acceptability of the research [44, 45]. There are also strong ethical arguments for public and patient involvement in decision-making about health and social care services and research [46, 47]. This is particularly important for those who are likely to be affected by the decisions, such as people with disabilities, vulnerable groups, and the elderly. Therefore, involving the patients in the design of the evaluation can help to ensure that research is designed and conducted in a way meaningful and relevant to people’s lives. The present study would benefit if patients had a more active role in the service evaluation planning. However, due to the fast-paced development of the service and the time constraints of this evaluation, patient involvement was not practiced in this research study. Further research involving patient input should be conducted to ensure the inclusion and activation of patients as partners at various stages of the research process. A replication of the study, including patient involvement would provide further credibility to the findings, as well as lead to further improvements in the field.

Overall, the present study aimed to explore the lived experiences and satisfaction of people receiving care in the step-down IC service, as part of a larger evaluation study of a step-down IC service in Buckinghamshire, UK. The findings in our study provide a useful overview of what was working well and what areas of improvement the service providers should focus on. The findings of this study can help inform the development of future step-down IC services and strengthen the quality of care provided.

## Strengths and limitations

The findings of this study add to a growing body of literature on IC. There is a lack of literature in IC that focuses on the patient experience – our study is one of the first to provide these insights, which will be invaluable in delivering patient-centred care. This study provides insights and guidance for policymakers and providers planning to implement and deliver intermediate care. The strength of this study lies in the fact that the data were generated in a real-life, step-down IC setting. Also, we conducted a rigorous evaluation using the intensive practices of thematic analysis away from the prevalent auditing techniques. The qualitative research checklist (COREQ) for reporting qualitative studies was completed.

One of the limitations of this research study is the small sample of conducted interviews and therefore low generalisability. A downside regarding our methodology is the analysis of the qualitative data by a single researcher due to limited resources. In addition, only 28 analysed questionnaires were returned to the research team and no information was given in relation to the total number of distributed questionnaires. Sample adequacy is an important consideration in evaluations and can impact the validity and generalizability of studies’ results. Therefore, it would be beneficial for future initiatives to keep a record of the distributed feedback questionnaires. It is important to highlight that the analysed data are related to the context of one step-down IC unit in Buckinghamshire, UK. The study commenced when the service had already stopped receiving new patients and had started to prepare for its closure. However, two different data sources were used to confirm that the collected data were robust. The use of data generated from the semi-structured interviews and the analysis of the questionnaires lead to confirmatory findings despite differences in methods of data collection, analysis, and interpretation.

Another limitation of this study is the limited involvement of the research team on the development of the patient feedback questionnaire. The questionnaire was developed by senior members of staff, who were involved in the implementation of the IC initiative. In the future, similar initiatives should involve the research team in the development of appropriate instruments to ensure good quality data and clear conclusions and recommendations. The use of a previously validated questionnaire is time and cost effective and allows the comparison of findings with those from similar studies. Health service research should encourage the use of validated questionnaires that can be compared across studies. A questionnaire that assesses patients’ satisfaction with the services will enable the service providers to identify areas where aspects of care could be improved [48].

## Conclusion

Overall, the participants appreciated the support they received from the step-down IC programme and felt encouraged to improve their functional abilities. Overall, patients were satisfied with the “person-centred” care in the step-down IC unit and they positively commented on the excellent relationships with the members of staff. Their rehabilitation plan aimed to increase their mobility and helped them to regain their independence. In this study, it was revealed that the patients were uninformed about their transfer to step-down IC and their package of care upon discharge. Therefore, a better hospital and IC discharge experience require members of staff to provide timely discharge information to the patients.

This evaluation study extends the current understanding of the patients’ lived experiences and satisfaction with care with step-down IC care. The findings of the study can be used as a frame of reference for the planning and implementation of future IC in the UK and elsewhere. As part of the process of improving quality it is crucial that IC services deliver patient-centred care, seeking the views and experiences of patients along the way.

## Electronic supplementary material

Below is the link to the electronic supplementary material.

Supplementary Material 1

Supplementary Material 2

Supplementary Material 3

Supplementary Material 4

## Data Availability

The data presented in this study are available on request from the corresponding author. The data are not publicly available due to reasons of privacy.

## Abbreviations

HAI: Healthcare-associated infection
IC: Intermediate care
NHS: National Health Service
NICE: National Institute for Health and Care Excellence
