# Supplementary figures and images for: Evaluating step-down, intermediate care programme in Buckinghamshire, UK: a mixed methods study
Source: BMC Public Health. 2023 Jun 6;23:1087. doi: 10.1186/s12889-023-15868-5 (PMC10242590; doi:10.1186/s12889-023-15868-5)

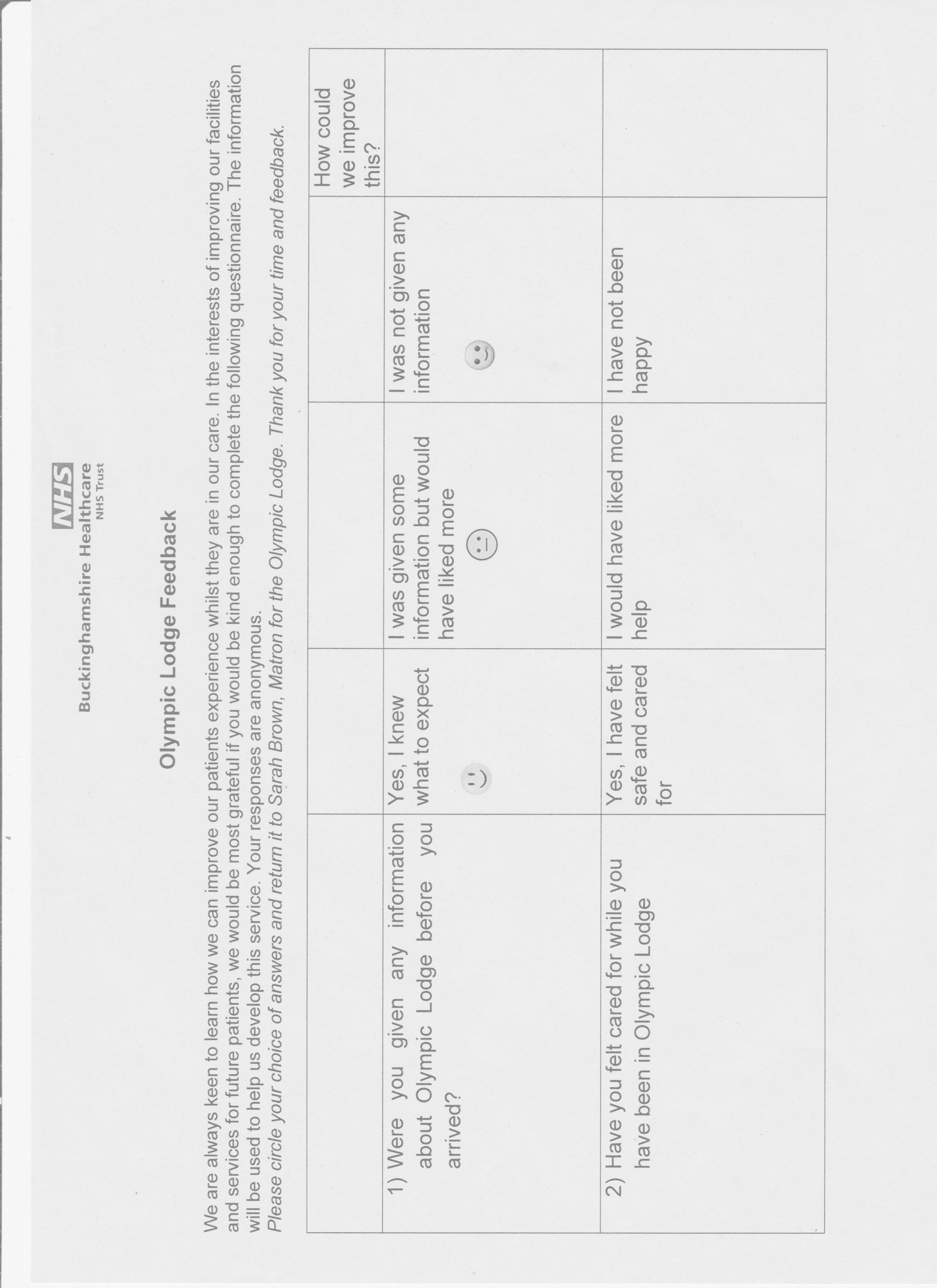

Supplement: Supplementary file 2 — Supplementary Material 2 [file 12889_2023_15868_MOESM2_ESM.jpg]

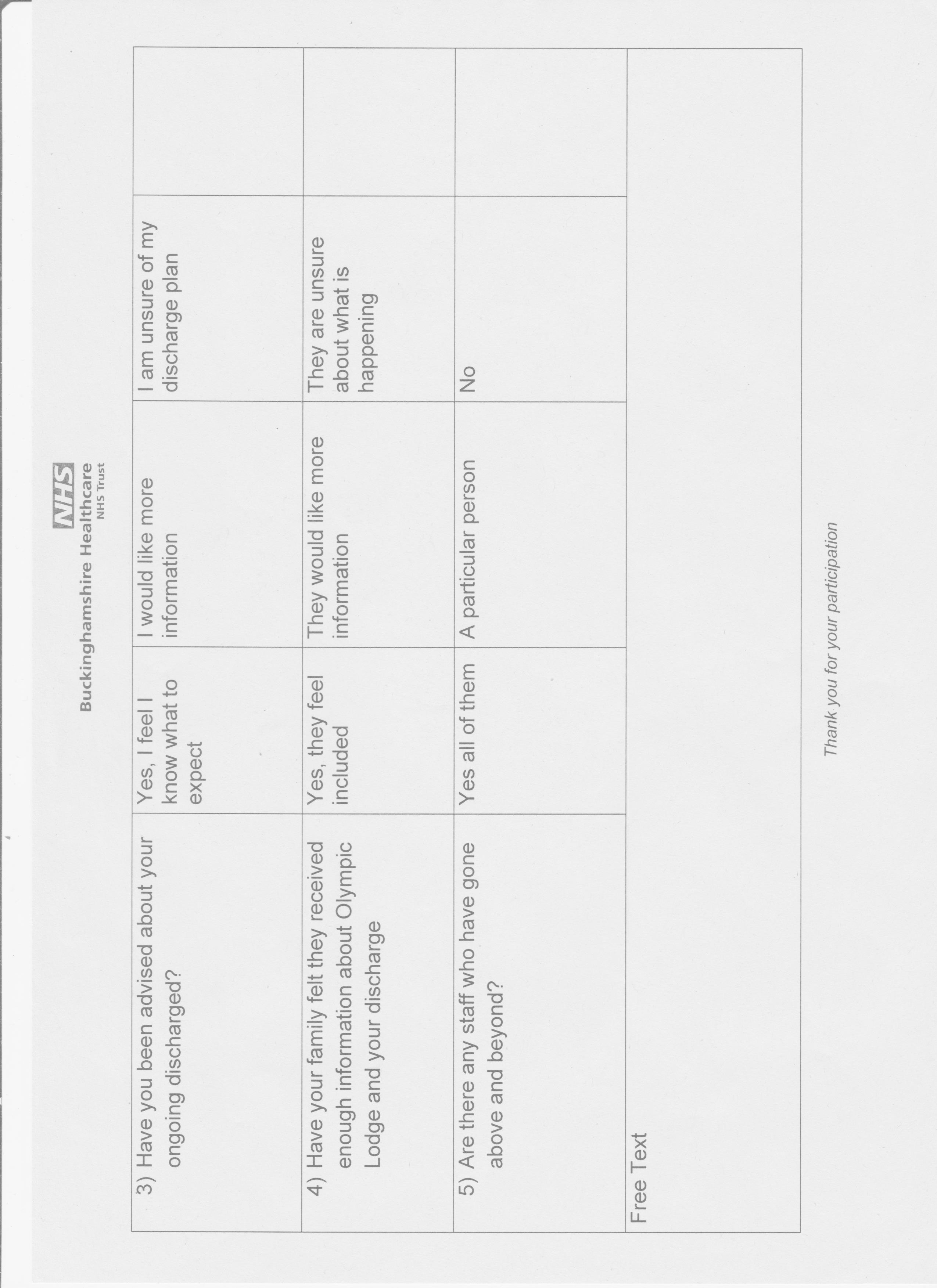

Supplement: Supplementary file 3 — Supplementary Material 3 [file 12889_2023_15868_MOESM3_ESM.jpg]
