# Supplementary material for: Evaluating step-down, intermediate care programme in Buckinghamshire, UK: a mixed methods study
Source: BMC Public Health. 2023 Jun 6;23:1087. doi: 10.1186/s12889-023-15868-5 (PMC10242590; doi:10.1186/s12889-023-15868-5)
Supplement: Supplementary file 4 — Supplementary Material 4 [file 12889_2023_15868_MOESM4_ESM.docx]

**Title of Project: Evaluating a step-down IC service : an exploration of patients’ experiences**

**Introduction**

- Thank participants for agreeing to take part in this interview.
- Provide background to the project
- Brief outline of the interview: We will discuss about your experiences from the service. There are no right or wrong answers.
- Explain about the consent procedure, highlight that the interview will be recorded, they do not have to answer any question they do not want to, stress that the participation in voluntary and they can stop the interview any time without giving any reason.
- The findings from the research will be written up in a report form.
- Confidentiality. Any quotes will be anonymised and I will ensure that there are no issues than can trace the interview with them.
- Before we start, participants will be asked to sign two copies of the consent form. One of them and one for the project’s file.
- Any questions or concerns before we start the interview?

**Interview schedule**

**Information**

I understand that you have been taken care of in the service. Do you remember whether you received any information about your move from the main hospital to the step-down service?

Prompt: If YES, do you remember what this was and who has given it to you?

Were you given the opportunity to ask questions?

**Transfer**

Do you remember the day you were transferred to the step-down service? Could you share with me what you remember? It is not a memory test. We just try to understand the patients’ experience.

Did you have to wait between the time that you were told that you would be transferred to the step-down service and the actual transfer?

**Staff**

How was it staying at the step-down service? How were the staff?

**Care**

How was your stay in step-down service?

Do you think it was a good idea to move you out of the hospital to the step-down service?

How do you feel after your stay at step-down service?

Prompt: do you feel more independent? / Confident?

**Ongoing plan**

Are you aware of a plan for your care?

Who spoke to you about it?

Do you feel you were given enough information?

Were you given the opportunity to ask questions?

**Family**

Have your family felt they received enough information about step-down service and your discharge?
